# Visualization of Midwives’ Professional Work Time a Comparative Analysis Between Obstetric Internal Medicine and Surgical Nursing

**DOI:** 10.7759/cureus.96702

**Published:** 2025-11-12

**Authors:** Chifumi Otaki, Kyoko Inoue, Kana Fujimoto, Ayumi Teraoka, Miki Nishikawa, Izumi Saito

**Affiliations:** 1 Graduate School of Medicine, Kyoto University, Kyoto, JPN; 2 Public Health, Kansai University of Health Sciences, Osaka, JPN; 3 Department of Nursing, Kobe University Hospital, Kobe, JPN; 4 Department of Nursing, University of Tokyo Health Sciences, Tokyo, JPN; 5 Department of Maternal Nursing, Tottori College of Nursing, Tottori, JPN; 6 Graduate School of Medicine, Kobe University, Kobe, JPN

**Keywords:** nursing management, patient safety, s: midwifery, staffing standards, time-motion study, workload

## Abstract

Background: Despite growing specialization, the domain-level structure of nurses’ and midwives’ daily activities remains under-characterized, and few studies compare obstetric with general wards using a single 37-subcategory taxonomy. In Japan, census- and acuity-based staffing under the Medical Care Act/DPC-PDPS captures headcount but underrepresents indirect care such as documentation and coordination. Quantifying domain-level time is therefore needed to align ward staffing with actual workload. We aimed to quantify obstetric midwives’ day-shift work time using the JNA 37 - subcategory framework and to compare domain and subcategory profiles, together with direct versus indirect care proportions, with internal medicine and surgical wards.

Methods: We conducted a prospective time-motion study in a Japanese university hospital. Midwives in the obstetric ward and nurses in internal medicine and surgical wards were continuously observed during weekday day shifts (08:30-17:15) using the Japanese Nursing Association’s 37-subcategory framework. Eligible staff were licensed midwives/nurses assigned to direct ward care for the full weekday day shift (08:30-17:15) who provided consent; exclusions were float/temporary, supernumerary/administrative, orientation-only, declined consent, and shifts curtailed by emergencies/extraordinary events. Three trained midwife observers performed continuous, one-to-one focal-follow observation, coding activities every 10 seconds with the JNA 37-subcategory taxonomy. To limit observation bias, observers rotated across staff and days, maintained an unobtrusive distance, and emphasized unit-level evaluation and de-identification during consent. The analytic unit was the day shift (obstetric 4 [29%], internal medicine 5 [36%], surgical 5 [36%]). For each domain and subcategory, minutes per shift were summarized as median (IQR). Domain-level differences were tested with Kruskal-Wallis, followed by Holm-adjusted Mann-Whitney pairwise tests. Subcategory differences were tested using one-way ANOVA with Games-Howell pairwise tests.

Results: Fourteen day shifts were analyzed. Ward differences were observed for Domain II (Kruskal-Wallis p = 0.0368) and Domain IV (p = 0.0200); only the Surgery vs. Internal Medicine contrast in Domain IV remained significant after Holm adjustment (p = 0.0476). Documentation & Coordination (Domain III) had the highest median in obstetrics, but the omnibus test did not reach significance (p = 0.0888).

Conclusions: Obstetric day shifts showed higher median time in Documentation & Coordination (Domain III), although the omnibus test was not significant; surgery had the highest median in Clinical Settings (Domain II; omnibus p = 0.0368); and Operational Management (Domain IV) differed by ward with only the Surgery vs. Internal Medicine contrast remaining significant after Holm adjustment (adjusted p = 0.0476). These patterns may inform staffing models that explicitly weight indirect-care tasks-especially documentation/coordination-alongside direct care when allocating resources. Future work should extend to nights/weekends and incorporate patient acuity and staffing mix. Generalizability is limited by the single-center, weekday day-shift design.

## Introduction

In increasingly specialized healthcare systems, nurses and midwives are expected to deliver autonomous, individualized, and evidence-based care, yet the distribution of their daily activities remains incompletely quantified [1]. Time-motion methodology provides a rigorous approach to describing work patterns and time allocation at the bedside, which is essential for aligning staffing with care needs and safeguarding quality [2]. International research further shows that appropriate nurse staffing is associated with lower mortality and better patient outcomes, underscoring the importance of measuring workload with sufficient granularity [3]. Within perinatal care, midwives’ expertise contributes to safer childbirth with fewer unnecessary interventions and improved maternal-newborn outcomes [4,5].

Despite this evidence, Japan’s current staffing framework under the Medical Care Act does not explicitly differentiate midwives from nurses in ward staffing standards, even though midwifery practice entails distinct responsibilities for maternal-neonatal safety, communication, and education [6]. Reports of prolonged working hours among obstetrics and gynecology staff further underscore the strain on perinatal services and the need for objective workload measurement [7]. Evidence from labor and delivery units during the COVID-19 pandemic also highlights missed critical nursing care processes, reinforcing the safety-critical role of coordination, documentation, and timely education in obstetric settings [8]. Prior time-motion studies on medical-surgical wards report substantial time devoted to documentation/coordination and preparation/medication workflows, which can constrain bedside exposure [9, 10]; however, few investigations have compared domain-level workload profiles across obstetric, internal medicine, and surgical wards using a common taxonomy. This gap raises a practical question: how does the structure of midwives’ work compare with that of nurses in general medical and surgical units?

Against this policy backdrop, our objective was to generate staffing-relevant evidence by producing domain- and subcategory-level time profiles for obstetric midwives using the JNA 37-subcategory framework, including summaries of direct versus indirect care, and by comparing these profiles with internal medicine and surgical wards to identify ward-specific patterns that can inform staffing and workflow improvements. Details of the statistical analyses are reported in the Methods.

## Materials and methods

We conducted a prospective observational time-motion study in a Japanese university hospital comprising one obstetric ward (26 beds, including six maternal-fetal intensive care beds), one surgical ward (51 beds; esophageal, gastrointestinal, gynecology), and one internal medicine ward (51 beds; rheumatology, nephrology, neurology, radiology). To capture routine operations and limit contextual heterogeneity, observations were restricted to weekday day shifts (08:30-17:15). Observation windows were January 2023 for the surgical ward, December 2023 for the internal medicine ward, and May 2023 for the obstetric ward.

Eligible participants were licensed midwives (obstetric ward) and registered nurses (internal medicine and surgical wards) who were rostered to deliver direct ward care for the full day shift (08:30-17:15) during the above windows and who provided written informed consent. We excluded float or temporary staff; supernumerary or purely administrative roles; orientation-only shifts; staff who declined participation; and shifts curtailed by emergencies or extraordinary events that precluded reliable observation. The unit of analysis was the individual day shift. In total, 14 shifts were observed - obstetric 4 (29%), internal medicine 5 (36%), and surgical 5 (36%). No patient-identifiable data were collected.

Three trained midwife observers conducted continuous, one-to-one focal-follow observation during weekday day shifts (08:30-17:15). Using a handheld timer, observers recorded the active subcategory at fixed 10-second intervals according to the Japanese Nursing Association’s nursing activity classification (1997), which comprises six domains and 37 subcategories (Table 1) [11]. Domain labels follow the JNA 37-subcategory taxonomy used in this study: I. Daily Living (1-11); II. Clinical Settings (12-19); III. Documentation & Coordination (20-22); IV. Operational Management (23-30); V. Organizational Management (31-34); VI. Other (35-37). Each interval received a single mutually exclusive code; task transitions were recorded at the next 10-second tick. When no direct task was occurring, the appropriate indirect or “other” category was assigned per the coding manual. Observers did not provide care, answer questions, or prompt actions. To avoid systematic pairing, observers rotated across staff and days according to a pre-specified schedule. For each observed shift and subcategory, we recorded cumulative duration in minutes and derived domain totals (Domains I-VI) as well as direct versus indirect care totals for contextual interpretation.

**Table 1:** Nursing activity framework (37 subcategories) English translation with minor adaptations (terminology harmonization and formatting) of the “Nursing Work Category Table (看護業務区分表)” from a Ministry of Health, Labour and Welfare meeting document (June 2002) (source: MHLW [11]). Reproduced under the MHLW Public Data License, Version 1.0 (attribution provided; changes indicated). MHLW: Ministry of Health, Labour and Welfare; NST: Non-Stress Test; EHR: Electronic Health Record; CAPD: continuous ambulatory peritoneal dialysis

Domain	Item No.	Activity (Subcategory)	Description (Details)
I. Daily Living	1	Eating/Feeding	Assistance with eating, posture/grooming, tube feeding, intake observation, tea service, meal setup/cleanup, and plating.
	2	Toileting/Elimination	Assistance with defecation/urination; posture/grooming; toilet walking assistance; diaper change; care during vomiting; stoma/catheter management; enema; urinary catheterization; suctioning of secretions.
	3	Hygiene/Cleanliness	Total/partial wipe; foot bath; hair wash/grooming; oral care; shaving/nail clipping; bathing/showering; face washing assistance; perineal wash; ear/nose care; change of patient clothes/linens; oshibori preparation; personal laundry organization.
	4	Safety	Prevention of falls/dangerous actions; monitoring/patrol; infection prevention (including MRSA); disaster prevention.
	5	Comfort/Relaxation	Turning patients, posture adjustment, external compresses, massage, psychological comfort (listening, being present, watching over).
	6	Inpatient Environment	Adjusting lighting/temperature; noise prevention; pest control; bed movement; bedside organization/cleanliness; bed making.
	7	Support for Independence	Patient education (diet, lifestyle, medication, injections, tests/procedures/surgery); rehabilitation (including phonation/respiration); bladder irrigation/training; CAPD; home nursing methods; counseling; recreation; orientation (admission, tests, pre-op, etc.).
	8	Patient Movement/Transfer	Assisted walking; wheelchair; stretcher transport (to OR, testing, X-ray, etc.).
	9	Patient and Family Communication	Contact with family; patient contact (telephone handling, messages); information exchange/consultation with family; nurse-call response; patient errands (e.g., shopping).
	10	End-of-Life Care	Watching over at the end of life; post-mortem care; attending physician–patient explanation; explaining procedures/forms; placement of remains; farewell support.
	11	Daily Living Prep/Cleanup	Preparation and cleanup for daily living assistance (tasks not requiring a nurse).
II. Clinical Settings	12	Receiving Orders/Reporting	Receiving orders, confirmation with the doctor, reporting patient condition, and doctor calls.
	13	Measurement/Vitals	Measuring T, P, R, BP; height; weight; chest/abdominal circumference; blood sugar; urine checks (e.g., glucose/ketone via test tape); CVP; level of consciousness; spirometry.
	14	Respiratory/Circulatory Management	Ventilator operation; oxygen tent/oxygen therapy; sputum promotion/suction; ultrasonic nebulizer; monitor observation (ECG); arterial line management; lung/heart auscultation; fluid balance check; NST monitoring.
	15	Medical Exam/Treatment Assistance	Rounds; bandage change; cast; blood transfusion/IV injection; IVH/continuous drip management; pre-/post-operative procedures; lavage; tests.
	16	Tests & Specimen Collection	Blood, urine, stool, sputum, gastric juice, bile, pleural/peritoneal fluid, CSF, tissue, secretions; endoscopy/catheter/X-ray tests, etc.
	17	Medication (Injection)	Subcutaneous and intramuscular injections.
	18	Medication (Non-Injection)	Oral; tube infusion; ointments; suppositories; eye/ear/nasal drops.
	19	Clinical Preparation/Cleanup	Dispensing; mixing; preparation/cleanup for procedures; specimen container/preparation/submission; results organization (tasks not requiring a nurse).
III. Documentation	20	Nursing Plans/Documentation	Admission nursing record; problem list; nursing plan; progress notes; temperature chart; summary; conference; EHR data collection.
	21	Other Records	Procedure plans; worksheet creation.
	22	Nurse-to-Nurse Handover	Handoff/report; contact between nurses (within the ward).
IV. Operational Management	23	External Communication	Contact with Pharmacy, Nutrition, Medical Affairs, Lab, Radiology, Outpatient, Central Supply, Accounting/Finance, and other wards/departments; viewing test data on computer (e.g., LIS/RIS). Note: Computer input, such as medication orders and diet communications, is included.
	24	Administrative Tasks	Admission/discharge guidance; patient lists; bed name tags; medication cards; meal slips; loan/checkout logs; chart organization; handling/organizing medical certificates and various slips; reception/liaison for visitors and guests.
	25	Supplies/Material Transport	Transport of supplies, documents, specimens, drugs, and other items; operation of transport equipment.
	26	Equipment/Instrument Management	Inspection of ventilators, ME equipment, crash carts, rounding carts, and cleaning/care carts.
	27	Non-Patient Room Environment	Organization of nurse station, staff room, procedure room, soiled utility room (etc.); maintenance/service requests.
	28	External Communication (Outside the Ward/Agencies)	Contact with Pharmacy, Nutrition, Medical Affairs, Lab, Radiology, Outpatient/Central Supply, Accounting/Finance, Nursing Department, other wards/departments; communication with public health centers and governmental authorities.
	29	Administrative Tasks (Registers & Forms)	Entries in admission/discharge ledgers; patient lists; bed name tags; medication cards; meal slips; procedure slips; loan/checkout logs; chart organization; handling/organizing medical certificates and various forms; reception/liaison for visitors and guests.
	30	Supplies/Material Transport (Equipment Operation)	Transport of supplies, documents, specimens, drugs, and other items; operation of transport machinery/equipment.
V. Organizational Management	31	Staff Duty and Adjustment	Creating duty rosters and weekly schedules; completing overtime orders; recording annual leave, etc.
	32	Guidance for Students/Staff	General guidance for nursing students; interviews; staff instruction; receiving instruction.
	33	Education/Training (OJT)	Participation in in-house training/study sessions.
	34	Meetings	Various committees/meetings; ward meetings.
VI. Other	35	Staff Health Management	Breaks/rest (including meals); health check-ups.
	36	Home Care Nursing	Home care nursing and other related general tasks.
	37	Other	Other general tasks.

To mitigate potential Hawthorne effects arising from consented, in-person observation, several steps were taken. First, participants were briefed that the study evaluated ward-level workflows rather than individual performance, and all records were de-identified. Second, observers maintained an unobtrusive position (approximately 1-2 meters), avoided conversation or prompts, and refrained from giving feedback during observation. Third, observers rotated across staff and days to prevent systematic pairing. Finally, a brief on-site acclimatization/orientation preceded coding so that staff could adjust to the observer’s presence. Residual observation bias is possible and is acknowledged in the Limitations.

Primary outcomes were minutes per day shift within each of the six domains and within each of the 37 subcategories. For contextual summaries, Items 1-19 were treated as direct care and Items 20-37 as indirect care in line with the JNA framework used. Secondary outcomes were minutes per day shift for direct versus indirect care. Continuous variables were summarized at the shift level as median (interquartile range, IQR). Categories with structural zeros (i.e., activity types not performed during specific shifts) were retained descriptively without inferential testing.

To mitigate confounding from nurse-to-patient ratios, elective procedure schedules, availability of ancillary services, and policy- or EHR-related workflow variation, we restricted observations to weekday day shifts, applied a single activity taxonomy across wards [11], used trained observers with standardized procedures, and rotated observers between wards. Daily bed occupancy and staffing context were documented descriptively for interpretation. Because the three wards were observed in different months, potential time/season effects (e.g., elective schedules, staffing patterns) may confound between-ward contrasts; this is addressed in the Limitations.

Between-ward differences in domain totals were tested with the Kruskal-Wallis test. When the omnibus test was significant, pairwise Mann-Whitney U tests with Holm adjustment were applied. For subcategory totals, we used one-way ANOVA; given unequal variances and unequal group sizes, pairwise comparisons employed the Games-Howell procedure. Statistical significance was set at two-sided p < 0.05. Because 37 subcategories were examined, subcategory-level findings are interpreted cautiously without across-item multiplicity correction. All analyses were performed at the shift level using IBM SPSS Statistics (version 31). Results are reported as median (IQR), with adjusted pairwise p values where applicable.

The study protocol was approved by the institutional review board (Approval No. B22014-H). All observed staff provided written informed consent prior to participation.

## Results

We analyzed 14 weekday day shifts (obstetrics 4 (29%), internal medicine 5 (36%), surgery 5 (36%)). The unit of analysis was the individual shift. Medians with IQRs are used for description, while hypothesis tests were conducted on shift-level raw minutes.

**Figure 1:**
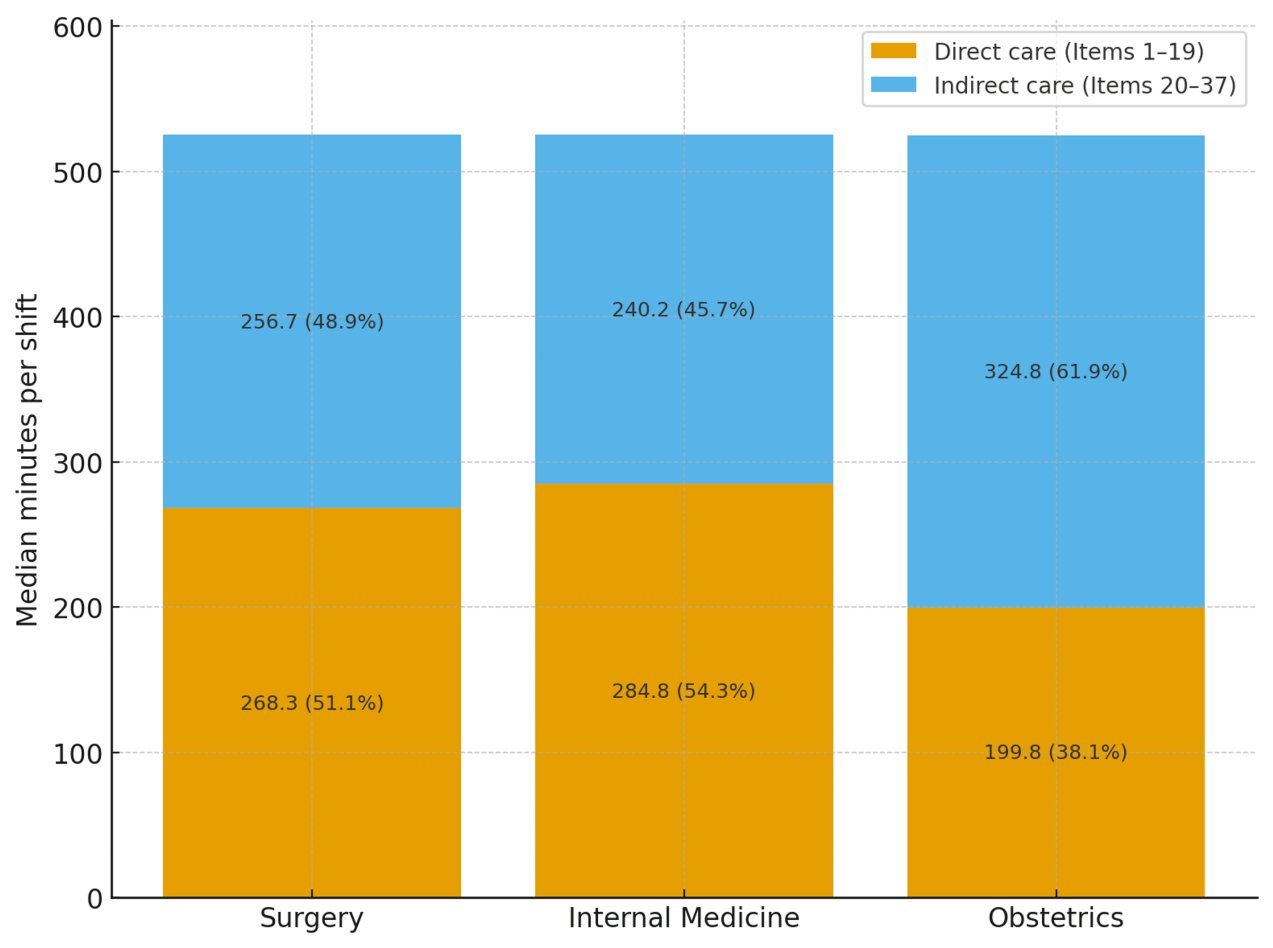
Direct vs. Indirect Care by Ward (Median Minutes per Day Shift) Stacked bars show median minutes per weekday day shift (08:30–17:15) spent in direct care (Items 1–19) and indirect care (Items 20–37) for Surgery, Internal Medicine, and Obstetrics (left to right). Values on bars indicate medians and percentages of total shift time. Data are shift-level medians; no statistical testing is applied in this figure (descriptive only).

**Figure 2:**
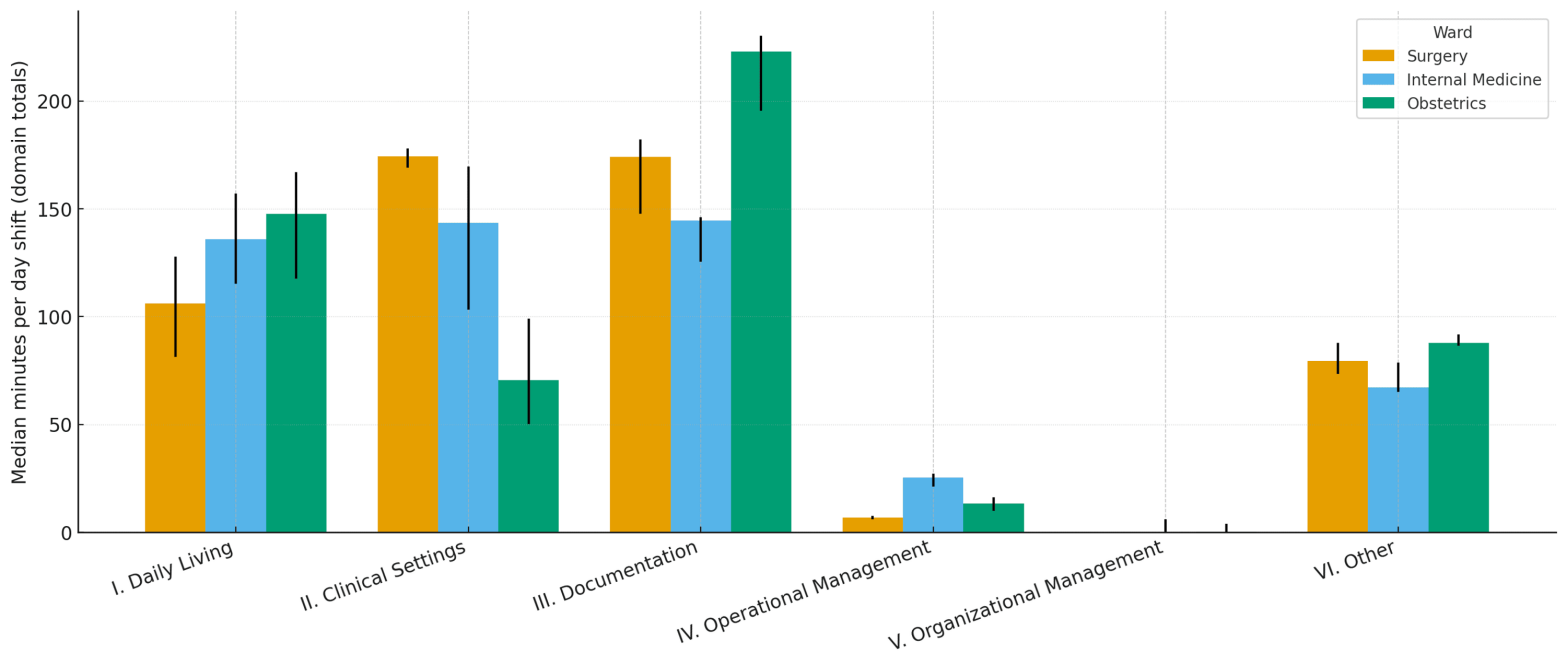
Domain-Level Time by Ward (Medians with IQRs) Median minutes per weekday day shift for the six JNA domains—I, Daily Living (1–11); II, Clinical Settings (12–19); III, Documentation & Coordination (20–22); IV, Operational Management (23–30); V, Organizational Management (31–34); VI, Other (35–37)—by Surgery, Internal Medicine, and Obstetrics. Error bars show interquartile ranges (IQRs). Omnibus Kruskal-Wallis tests indicated differences for Domain II (p = 0.0368) and Domain IV (p = 0.0200); after Holm adjustment, only the Surgery vs. Internal Medicine contrast in Domain IV remained significant (adjusted p = 0.0476). Domain III had the highest median in Obstetrics, but the omnibus test was not significant (p = 0.0888). Other domains are descriptive.

Figure 1 shows that obstetrics devoted a larger share of time to indirect care, whereas surgery and internal medicine were predominantly direct-care oriented. At the domain level (Figure 2), between-ward differences were observed for Clinical Settings (Domain II) on the Kruskal-Wallis test (p=0.0368), with surgery exhibiting the greatest median, and for Operational Management (Domain IV) (p=0.0200). After Holm-adjusted Mann-Whitney pairwise testing, only the surgery versus internal medicine contrast in Domain IV remained significant (adjusted p=0.0476; indicated by the bracket with an asterisk in Figure 2). Documentation & Coordination (Domain III) had the highest median in obstetrics, yet the omnibus test did not reach significance (p=0.0888). Other domains showed descriptive differences without significant omnibus effects.

**Figure 3:**
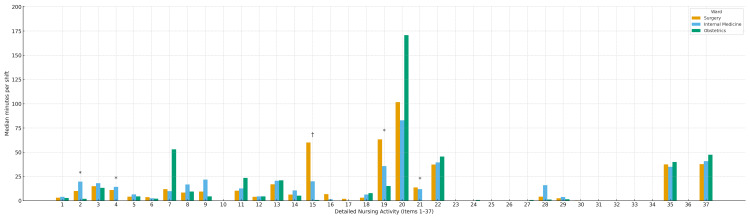
Subcategory-Level Time (37 Items) by Ward (Medians) Bars show median minutes per weekday day shift for each JNA subcategory (Items 1–37) in Surgery, Internal Medicine, and Obstetrics. One-way ANOVA was used for omnibus screening, followed by Games–Howell pairwise comparisons. Asterisks (*) denote pairwise differences that remained significant after Games–Howell for Items 2, 4, 19, and 21 (all p < 0.05). A dagger (†) marks an exploratory contrast for Item 15 (Surgery > Obstetrics) observed despite a non-significant omnibus test (p = 0.102); interpret cautiously given small sample size and multiple testing. All other subcategory contrasts were not significant. No across-item multiplicity correction was applied; subcategory findings are hypothesis-generating.

At the subcategory level (Figure 3; Table 2), one-way ANOVA detected omnibus differences in 8 of 37 subcategories (Items 2, 4, 7, 9, 19, 20, 21, and 28; p < 0.05). Using Games-Howell for pairwise comparisons (allowing unequal variances and unequal n), significant contrasts persisted only for Items 2, 4, 19, and 21 (all p < 0.05). Specifically, Item 2 was higher in surgery than obstetrics; Item 4 was higher in surgery than obstetrics and in internal medicine than obstetrics; Item 19 was higher in surgery than obstetrics and in internal medicine than obstetrics, with surgery versus internal medicine not significant; and Item 21 was higher in surgery than obstetrics and in internal medicine than obstetrics. Item 15 showed a surgery-greater-than-obstetrics contrast on Games-Howell despite a non-significant omnibus ANOVA (p = 0.102) and is therefore reported exploratorily without inferential claims. For all remaining items, no pairwise differences reached significance on Games-Howell. Full medians (IQRs) and Games-Howell adjusted p-values are provided in Table 2.

**Table 2:** Time per shift by nursing activity subcategory across wards Values are minutes per shift. Data are shown as Mean ± SD and Median (IQR) for each ward. Sample sizes: surgery n=5, internal medicine n=5, obstetrics n=4. Subcategory numbering follows the Japanese Nursing Association Nursing Activity Classification (1997). SD, standard deviation; IQR, interquartile range. Between-ward differences were examined with one-way ANOVA and Games–Howell post hoc tests; statistically significant results are interpreted in the Results section. Structural zeros indicate activities not observed during the weekday day shifts studied.

Item No.	Subcategory	Surgery: Mean ± SD	Surgery: Median (IQR)	Internal Medicine: Mean ± SD	Internal Medicine: Median (IQR)	Obstetrics: Mean ± SD	Obstetrics: Median (IQR)
1	Eating/Feeding	3.23 ± 1.49	3.17 [3.17–3.67]	7.17 ± 7.19	4.00 [3.00–8.33]	2.71 ± 2.07	2.92 [1.33–4.08]
2	Toileting/Elimination	11.47 ± 3.17	10.00 [9.17–12.67]	16.93 ± 11.76	19.67 [5.00–25.00]	2.42 ± 2.87	2.00 [0.00–4.83]
3	Hygiene/Cleanliness	14.90 ± 11.85	15.00 [5.50–24.17]	17.70 ± 16.87	18.17 [7.00–19.00]	16.42 ± 15.82	13.33 [5.67–27.17]
4	Safety	12.27 ± 6.14	11.00 [5.50–12.67]	14.37 ± 4.78	12.67 [11.50–18.50]	1.38 ± 2.75	0.00 [0.00–2.75]
5	Comfort/Relaxation	8.03 ± 6.54	4.17 [3.17–13.00]	9.93 ± 5.52	8.67 [5.00–13.17]	5.33 ± 3.77	4.42 [2.58–8.08]
6	Inpatient Environment	4.30 ± 2.01	3.50 [2.67–5.50]	4.77 ± 4.82	4.08 [2.00–7.92]	3.75 ± 3.76	2.08 [1.50–6.00]
7	Support for Independence	17.37 ± 12.87	11.17 [5.83–26.33]	9.33 ± 7.09	11.17 [0.83–12.33]	53.96 ± 34.52	53.00 [25.67–82.25]
8	Patient Movement/Transfer	7.77 ± 8.28	8.50 [0.00–10.50]	19.60 ± 6.60	16.83 [14.00–25.67]	19.54 ± 20.99	9.42 [8.42–30.67]
9	Patient and Family Communication	11.77 ± 5.20	9.50 [8.17–14.33]	23.50 ± 13.20	21.83 [20.33–22.50]	5.67 ± 4.21	4.42 [2.58–8.75]
10	End-of-Life Care	0.00 ± 0.00	0.00 [0.00–0.00]	0.00 ± 0.00	0.00 [0.00–0.00]	0.00 ± 0.00	0.00 [0.00–0.00]
11	Daily Living Prep/Cleanup	14.60 ± 10.40	11.83 [6.67–19.50]	14.80 ± 5.88	14.50 [10.92–19.00]	25.67 ± 6.68	24.83 [21.67–31.00]
12	Receiving Orders/Reporting	4.13 ± 2.99	3.17 [2.00–5.33]	6.73 ± 5.69	5.00 [2.83–8.25]	5.17 ± 3.20	4.33 [2.50–7.62]
13	Measurement/Vitals	19.93 ± 10.09	17.00 [9.83–27.17]	21.27 ± 10.44	22.50 [14.50–29.17]	23.83 ± 17.02	17.33 [9.38–38.00]
14	Respiratory/Circulatory Management	9.87 ± 5.77	9.83 [5.00–14.25]	11.73 ± 6.89	12.50 [9.75–13.92]	12.33 ± 18.35	3.88 [0.00–25.42]
15	Medical Exam/Treatment Assistance	57.97 ± 5.06	58.33 [56.67–61.92]	39.80 ± 48.15	23.00 [9.83–55.92]	9.29 ± 17.59	2.17 [0.00–19.38]
16	Tests & Specimen Collection	6.77 ± 6.23	4.83 [1.17–11.50]	14.77 ± 23.83	3.75 [0.00–23.50]	0.00 ± 0.00	0.00 [0.00–0.00]
17	Medication (Injection)	1.97 ± 1.61	2.00 [0.50–3.50]	0.77 ± 1.23	0.50 [0.00–1.75]	2.00 ± 4.00	0.00 [0.00–4.00]
18	Medication (Non-Injection)	4.73 ± 3.69	4.33 [1.50–7.92]	7.40 ± 4.88	6.67 [4.67–10.25]	7.38 ± 5.36	7.29 [3.88–10.67]
19	Clinical Preparation/Cleanup	65.07 ± 18.90	59.67 [51.83–85.83]	40.03 ± 8.21	39.67 [34.83–47.34]	18.58 ± 10.14	16.50 [12.25–24.96]
20	Nursing Plans/Documentation	106.97 ± 17.24	104.50 [92.58–118.17]	87.17 ± 17.92	82.33 [72.92–97.58]	158.54 ± 49.51	145.23 [99.06–192.67]
21	Other Records	16.53 ± 8.75	13.33 [9.25–26.33]	13.30 ± 5.16	12.50 [10.00–17.17]	0.00 ± 0.00	0.00 [0.00–0.00]
22	Nurse-to-Nurse Handover	38.97 ± 10.67	39.00 [31.50–45.00]	39.80 ± 8.78	40.83 [34.33–46.33]	44.29 ± 10.30	41.25 [35.00–53.00]
23	External Communication	0.00 ± 0.00	0.00 [0.00–0.00]	0.20 ± 0.45	0.00 [0.00–0.50]	0.00 ± 0.00	0.00 [0.00–0.00]
24	Administrative Tasks	0.00 ± 0.00	0.00 [0.00–0.00]	0.00 ± 0.00	0.00 [0.00–0.00]	2.58 ± 4.19	0.00 [0.00–4.50]
25	Supplies/Material Transport	0.00 ± 0.00	0.00 [0.00–0.00]	0.70 ± 1.57	0.00 [0.00–1.00]	0.00 ± 0.00	0.00 [0.00–0.00]
26	Equipment/Instrument Management	0.00 ± 0.00	0.00 [0.00–0.00]	0.00 ± 0.00	0.00 [0.00–0.00]	0.00 ± 0.00	0.00 [0.00–0.00]
27	Non-Patient Room Environment	0.37 ± 0.51	0.33 [0.00–0.83]	0.37 ± 0.51	0.33 [0.00–0.83]	3.58 ± 5.73	2.38 [0.88–4.88]
28	Clinical Communication (Calls/Coordination outside ward)	4.03 ± 1.42	4.00 [3.00–4.50]	18.13 ± 9.99	16.00 [9.58–29.00]	3.92 ± 5.85	2.19 [0.88–4.94]
29	Errands/Administrative Liaison (Patient-related)	2.27 ± 0.80	2.33 [1.83–2.67]	4.13 ± 2.62	4.17 [2.58–5.83]	1.92 ± 2.01	2.00 [0.00–3.42]
30	Supplies Check/Ordering (Minor)	0.37 ± 0.41	0.33 [0.17–0.67]	0.10 ± 0.15	0.08 [0.00–0.17]	0.75 ± 1.50	0.00 [0.00–1.50]
31	Staff Duty and Adjustment	0.00 ± 0.00	0.00 [0.00–0.00]	0.00 ± 0.00	0.00 [0.00–0.00]	2.38 ± 4.42	0.00 [0.00–2.25]
32	Guidance for Students/Staff	0.00 ± 0.00	0.00 [0.00–0.00]	3.00 ± 4.24	1.00 [0.00–6.00]	0.00 ± 0.00	0.00 [0.00–0.00]
33	Education/Training (OJT)	0.00 ± 0.00	0.00 [0.00–0.00]	0.00 ± 0.00	0.00 [0.00–0.00]	1.25 ± 2.50	0.00 [0.00–1.25]
34	Meetings	0.00 ± 0.00	0.00 [0.00–0.00]	0.00 ± 0.00	0.00 [0.00–0.00]	0.00 ± 0.00	0.00 [0.00–0.00]
35	Staff Health Management	39.67 ± 8.18	39.00 [35.50–42.67]	34.03 ± 9.01	33.33 [27.08–41.50]	38.75 ± 4.79	38.75 [35.25–42.63]
36	Home Care Nursing	0.00 ± 0.00	0.00 [0.00–0.00]	0.00 ± 0.00	0.00 [0.00–0.00]	0.00 ± 0.00	0.00 [0.00–0.00]
37	Other	39.70 ± 8.11	40.00 [34.00–45.00]	43.47 ± 13.41	42.00 [33.67–52.67]	51.38 ± 10.60	51.25 [46.00–58.00]

Given the small number of observed shifts and multiple testing across 37 subcategories (no multiplicity correction across items), the subcategory findings should be interpreted cautiously and primarily as descriptive signals to guide future confirmation.

## Discussion

This comparative time-motion study visualizes midwives’ professional work within a standardized 37-subcategory framework [11] and is conceptually aligned with the midwifery quality-of-care framework emphasizing coordination, education, and respectful, woman-centered care [12]. It compares domain-level profiles with internal medicine and surgical wards. Consistent with the Results, ward differences were detected for Clinical Settings (Domain II) and for Operational Management (Domain IV; including patient education/guidance subcategories). After Holm adjustment, only the Surgery-versus-Internal Medicine contrast in Domain IV remained statistically significant. Documentation & Coordination (Domain III) showed the highest median in obstetrics; however, the omnibus test did not reach significance. Accordingly, we interpret a comparatively greater indirect-care burden in obstetrics as a suggestive pattern rather than a confirmatory finding, while recognizing that medical-surgical wards appear to allocate a larger share to operational direct-care tasks.

Several institutional features may plausibly shift domain allocations across wards. Foundational quality-and-safety literature underscores the importance of appropriate staffing and skill-mix for care processes [1], and experience mix (e.g., higher novice proportion, rotating trainees) can increase supervisory/coordination time [7]. Patient acuity and case-mix (e.g., operative/induction rates, comorbidity burden) are likely to elevate surveillance and escalation demands, influencing Domains I-II; perinatal demand dynamics documented in a tertiary setting support this interpretation [13]. Electronic documentation design and local policies (template structure, required checklists, signature rules) are known to shape Domain III workload and can divert time from bedside care when usability is suboptimal [9,10,14]. Conversely, streamlined templates and team-based documentation may release time to direct care. Procedure density and ancillary support (e.g., procedure assistants, ward clerks) can raise or buffer Domain II demands. Unit layout (travel distances) and handover norms (frequency/structure) further modulate Domains II-IV. These contextual levers help explain the observed differences and indicate where local redesign (EHR templates, assistant roles, standardized handovers) may be most impactful [1,4,12,14].

Within perinatal services, documentation/coordination and family-inclusive education are central to safety, aligning with the higher obstetric medians we observed for Domains III-IV, even though Domain III did not achieve statistical significance [4,5,12,15]. Our patterns are directionally consistent with prior ward-based time-motion reports in general units that describe substantial documentation/coordination and preparation/medication workload, potentially constraining bedside exposure [9,10,14]. Differences in magnitude across studies likely reflect documentation policies and EHR usability, staffing models and experience mix, procedure schedules, and local workflow design factors that can moderate domain shares between settings [1,7,13,14]. Given the observational design and limited sample, we refrain from causal claims and position our findings as context-dependent descriptions that can guide hypothesis generation [2].

Practical implications should therefore be framed as locally testable hypotheses rather than prescriptive rules. In surgery, where Domain II (Clinical Settings) demand is high, planned coverage for procedure assistance and smoothing of preparation/cleanup (Item 19) may be evaluated. In obstetrics, staffing and skill-mix may consider explicitly accommodating Documentation & Coordination load (Domain III)-for example, protected time or clerical/assistant support during peak periods-while monitoring whether such measures preserve bedside availability. Across wards, standardizing handovers and simplifying EHR templates may reduce duplication within high-load subcategories (Domains III-IV), potentially releasing time to direct care. We recommend routine monitoring of minutes-per-shift by domain (I-VI) to evaluate the impact of such adjustments and to support iterative quality improvement. In Japan, where staffing indicators remain largely census/acuity-oriented, incorporating domain-level metrics, especially for indirect care, could complement existing indices when interpreting local staffing needs within the Japan Staffing Framework [11].

We sought to limit confounding by restricting observations to weekday day shifts, using a single activity taxonomy across wards, and standardizing observer procedures; nonetheless, residual confounding from unmeasured factors (e.g., acuity, nurse-to-patient ratios, experience mix, EHR/policy design, and month-to-month operational variation) cannot be excluded. In particular, observations occurred in different months across wards, so time/season effects (e.g., elective schedules and staffing patterns) may have influenced between-ward contrasts. Given the modest number of observed shifts, some effects-particularly where omnibus significance did not translate into adjusted pairwise differences-may be underpowered or attenuated by heteroscedasticity. Because 37 subcategories were examined, we did not apply across-item multiplicity correction; hence, subcategory findings are hypothesis-generating. Future studies should extend to nights/weekends and multiple sites, incorporate shift-level covariates (ratios, procedure counts, case-mix indices, documentation workload), and apply multilevel models to separate within- and between-ward variance. Linking domain/subcategory profiles to patient-level outcomes would further clarify practical relevance and support evidence-informed refinements to staffing guidance.

## Conclusions

This comparative time-motion study suggests that obstetric day shifts may carry a comparatively greater indirect-care workload, especially documentation and coordination, while surgical wards appear to devote a larger share to treatment and procedures, with internal medicine intermediate between these patterns. Because domain-level differences were limited after multiplicity adjustment (and Domain III did not reach omnibus significance), practice implications should be framed as locally testable hypotheses. Specifically, hospitals may consider: (i) explicitly accounting for indirect-care load in obstetric staffing (e.g., protected time and/or assistant/clerical support for documentation and handovers); (ii) streamlining EHR and handover workflows to reduce duplication and potentially release bedside time; (iii) ensuring adequate procedure-assist coverage in surgery to match peak procedural demand; and (iv) routine monitoring of domain totals (Domains I-VI) to evaluate and iteratively adjust staffing and workflows. Multi-site studies spanning nights/weekends and integrating acuity, staffing mix, and patient outcomes are warranted to confirm generalizability and quantify impact.
